# Quantitative MRI Assessment Using Variable Echo Time Imaging of Peripheral Nerve Injury in ATTRv Amyloidosis Patients

**DOI:** 10.1111/ene.70172

**Published:** 2025-04-23

**Authors:** Carlo Asteggiano, Matteo Paoletti, Elisa Vegezzi, Xeni Deligianni, Francesco Santini, Niels Bergsland, Nico Papinutto, Massimiliano Todisco, Giuseppe Cosentino, Andrea Cortese, Laura Obici, Giovanni Palladini, Anna Pichiecchio

**Affiliations:** ^1^ Department of Brain and Behavioral Sciences University of Pavia Pavia Italy; ^2^ Advanced Imaging and Artificial Intelligence Center IRCCS Mondino Foundation Pavia Italy; ^3^ IRCCS Mondino Foundation Pavia Italy; ^4^ Basel Muscle MRI. Department of Biomedical Engineering University of Basel Basel Switzerland; ^5^ Buffalo Neuroimaging Analysis Center, Department of Neurology Jacobs School of Medicine and Biomedical Sciences, University at Buffalo, The State University of New York Buffalo New York USA; ^6^ Weill Institute for Neurosciences, Department of Neurology University of California San Francisco San Francisco California USA; ^7^ Translational Neurophysiology Research Unit IRCCS Mondino Foundation Pavia Italy; ^8^ Amyloidosis Research and Treatment Center Foundation IRCCS Policlinico San Matteo Pavia Italy; ^9^ Department of Molecular Medicine University of Pavia Pavia Italy

**Keywords:** ATTRv, biomarker, magnetic resonance imaging (MRI), polyneuropathy, transthyretin amyloidosis

## Abstract

**Background and Purpose:**

Early detection of peripheral nerve damage in patients with hereditary transthyretin amyloidosis (ATTRv) has become essential for the prompt initiation of effective, recently approved therapies. In our study, we propose a new variable echo time (vTE) MRI sequence as a non‐invasive method to detect nerve injury in ATTRv patients and to establish a novel potential imaging marker of neuropathy that correlates with disease severity and abnormal results of NCS.

**Methods:**

In this cohort study, twenty patients with clinically confirmed ATTRv polyneuropathy (PNP) and twenty‐one healthy volunteers underwent 3 T MRI. vTE was performed on the right thigh to include the proximal tract of the sciatic nerve. The cross‐sectional area of the whole sciatic nerve, inner epineurium, and endoneurial fascicles was segmented, and the corresponding pseudo‐T2* was extrapolated from the two acquired echoes of the vTE.

**Results:**

Significantly higher fascicles pT2* (*p* = < 0.001), total cross‐sectional area (CSA: *p* = 0.017) and fascicular area (*p* = < 0.001) were found in the ATTRv group compared to healthy controls. Fascicles pT2* also correlated with previously validated clinical outcome measures such as Polyneuropathy Disability Scoring System (PND score *p* = < 0. 001), Neuropathy Impairment Score (NIS *p* = 0.030) and NIS items related to the lower limbs, and with nerve conduction parameters, demonstrating the ability to discriminate ATTRv patients with different degrees of PNP from HC.

**Conclusion:**

In conclusion, the vTE sequence provides novel and reliable imaging markers capable of detecting early nerve microstructural changes related to disease onset and severity.

## 1 Introduction

Hereditary transthyretin amyloidosis (ATTRv) is an uncommon yet severe, systemic disease resulting from the accumulation of a mutated form of transthyretin (TTR), a physiological transporter of serum thyroxine and retinol‐binding protein. Mutations in the TTR gene on chromosome 18 cause protein misfolding, leading to abnormal amyloid fibril deposition in various organs, notably the heart and peripheral nerves. The clinical presentation of ATTRv typically includes progressive sensorimotor polyneuropathy, autonomic dysfunction, and cardiac failure, with a mean survival of 7–10 years after onset if left untreated [1, 2, 3, 4, 5, 6].

ATTRv polyneuropathy (ATTRv‐PNP) stems from the accumulation of misfolded TTR in the peripheral nervous system (PNS), resulting in length‐dependent neuropathy [4, 7]. Pathologically, amyloid fibril deposition initiates in the dorsal root ganglia and autonomic ganglia, where the blood‐nerve barrier is more permeable [8]. Subsequently, TTR deposition progresses along the peripheral nerve, following a proximal‐to‐distal gradient in line with the axoplasmic endoneurial flow [9, 10]. Amyloid fibrils and prefibrillar material predominantly accumulate around endoneurial capillaries, causing extensive microstructural changes and widespread axonal loss within the nerve [11, 12, 13].

The typical diagnostic work‐up in ATTRv‐PNP patients includes a neurological examination coupled with nerve conduction studies (NCS). However, these methods have limitations in detecting early nerve lesions and determining the site and extent of lesions [14]. With various therapies recently approved for ATTRv amyloidosis treatment and others in development, there is an ongoing need for sensitive and reliable biomarkers and imaging markers to aid in disease onset detection, progression tracking, and drug efficacy monitoring [15, 16, 17, 18, 19, 20, 21, 22, 23].

Magnetic resonance neurography (MRN) has the potential to overcome diagnostic limitations by directly identifying nerve injuries in several peripheral neuropathies, including ATTRv‐PNP [24, 25, 26, 27, 28, 29, 30, 31]. Different quantitative MRI parameters, such as apparent T2 relaxation time (T2app), normalized signal intensity (NSI), and spin proton density (*ρ*), have been shown to detect microstructural changes in peripheral nerves mainly due to an increase in free and unbound water caused by inflammation [32, 33, 34, 35]. Diffusion tensor imaging (DTI) and magnetization transfer imaging with the easily computable magnetization transfer ratio (MTR), a semi‐quantitative parameter sensitive to myelin changes in various peripheral and central neurological diseases, have recently been shown to identify macromolecular changes and microstructural alterations in nerves of patients affected by ATTRv amyloidosis and other neuropathies [14, 31, 36, 37, 38, 39, 40, 41, 42].

In our study, we propose an innovative application of MRN using a dual echo variable echo time (vTE) sequence. Due to its very short echo time (TE), this sequence enables the quantification of the signal from short T2 relaxation time components of the nerve, including myelin and epineurium. It also allows for signal suppression of long T2 relaxation time components in the nerve, such as small unbound water molecules that increase as a consequence of inflammation and endoneurial edema [43].

The higher in‐plane spatial resolution of the chosen vTE protocol, when compared to standard MTR and DTI protocols, allows for a better visualization of the different components of the nerve, primarily represented by the inner epineurium and fascicles. This feature enables the extrapolation of morpho‐volumetric measurements and helps distinguish the contributions of these different structures to the overall pT2* signal.

Our study aims to evaluate the application of the vTE sequence as a non‐invasive method to detect nerve injury in ATTRv patients and to establish a novel potential imaging marker of neuropathy that correlates with disease severity and abnormal results from NCS.

## 2 Methods

### 2.1 Study Design and Patients

This prospective cross‐sectional study involving human participants adhered to the ethical standards of the institutional and/or national research committee and the 1964 Helsinki Declaration and its later amendments or comparable ethical standards. The study was approved by the Ethic Committee of IRCCS Fondazione Policlinico S. Matteo (p‐20170020469) and by local institutional review boards. All subjects gave their written informed consent to participate.

Twenty patients with confirmed ATTRv were enrolled between September 2017 and August 2018 at the Amyloidosis Research and Treatment Center within the University of Pavia in Pavia, Italy. Key enrollment criteria included the presence of a documented mutation either by Sanger sequencing or targeted panel in the *TTR* gene (*Glu89Gln, Ile68Leu, Phe64Leu, Tyr78Phe, Val30Met, Thr49Ala, Ala109Ser*). Patients with diabetes mellitus, alcohol abuse, any malignancy, or infectious disease, all of which represent risk factors for polyneuropathy, were excluded from the study. Twenty‐one healthy controls (HCs), matched for age and sex, were also enrolled. Exclusion criteria for all participants included pregnancy and MRI safety‐related contraindications. All subjects gave their written informed consent to participate.

All patients underwent detailed assessment by two neurologists (E.V. and A.C., with 5 and 10 years of experience, respectively), including demographic records, past medical history, and full neurologic examination. Patients were rated using the Polyneuropathy Disability scoring system (PND‐score), Neurologic Impairment Score (NIS), and NIS for the Lower Limbs (NIS‐LL) [44, 45, 46]. PND score was graded as follows: PND = 1 (sensory disturbances with preserved walking capability), PND = 2 (sensory‐motor symptoms with unassisted gait), PND = 3 (sensory‐motor symptoms with assisted gait), and PND = 4 (wheelchair‐bound or bedridden). ATTRv‐PNP patients were defined as symptomatic when PND scored ≥ 1.

Based on the individual NIS‐LL (NIS‐LL Grade), PNP severity was further classified as mild (NIS‐LL 1–20), moderate (NIS‐LL 21–61), or severe (NIS‐LL 62–88) [33, 34]. A subset of 14 patients underwent nerve NCS at the same time as MRI, and motor conduction of the tibial and peroneal nerves (i.e., distal motor latency, nerve conduction velocity, NCV, and compound muscle action potential, CMAP) together with sensory conduction of the sural nerve (SNAP) was evaluated in all cases. The patients were classified into two groups based on NCS findings: axonal and axonal with reduced conduction velocity.

### 2.2 MRI Acquisition

All participants underwent MRI scanning on a Siemens 3 T Skyra scanner using either an 8‐channel surface coil or a 32‐channel spine coil [43, 44]. Our protocol included a 3D monopolar dual echo vTE sequence, previously validated in a cohort of healthy subjects (TE_1_/TE_2_ = 1.05/5.37 ms, TR = 19 ms, flip angle = 10°, Field‐of‐view = 290 mm × 236 mm, voxel size = 0.6 × 0.6 × 5.0 mm^3^, number of slices = 12, acquisition time = 5 min 40 s) [43, 44]. The longitudinal coverage of the sciatic nerve was 6 cm. Binomial excitation was used to suppress the fat signal. The first echo image was acquired with an asymmetric variable echo time, while the second was fully sampled. The two echoes are sensitive to long T2* species; however, short T2* species are sampled mainly by the first echo and their signal reduces considerably at the time of the second echo.

### 2.3 Image Analysis

For signal quantification, the pseudo‐T2* (pT2*) was calculated as an indication of T2*, which is known to be a quantifiable parameter strictly related to the intrinsic T2 relaxation time of a specific tissue, thus reflecting the microstructural composition in vivo, according to the following formula:

$$\text{pseudo}-T_{2}*=\frac{-\Delta \mathrm{TE}}{\ln\left(\frac{\mathrm{iTE}2}{\mathrm{iTE}1}\right)}$$

iTE2 and iTE1 are the signal intensities of the second and first echo images, respectively, and ΔTE is the difference between the two echo times [43, 44].

**FIGURE 1:**
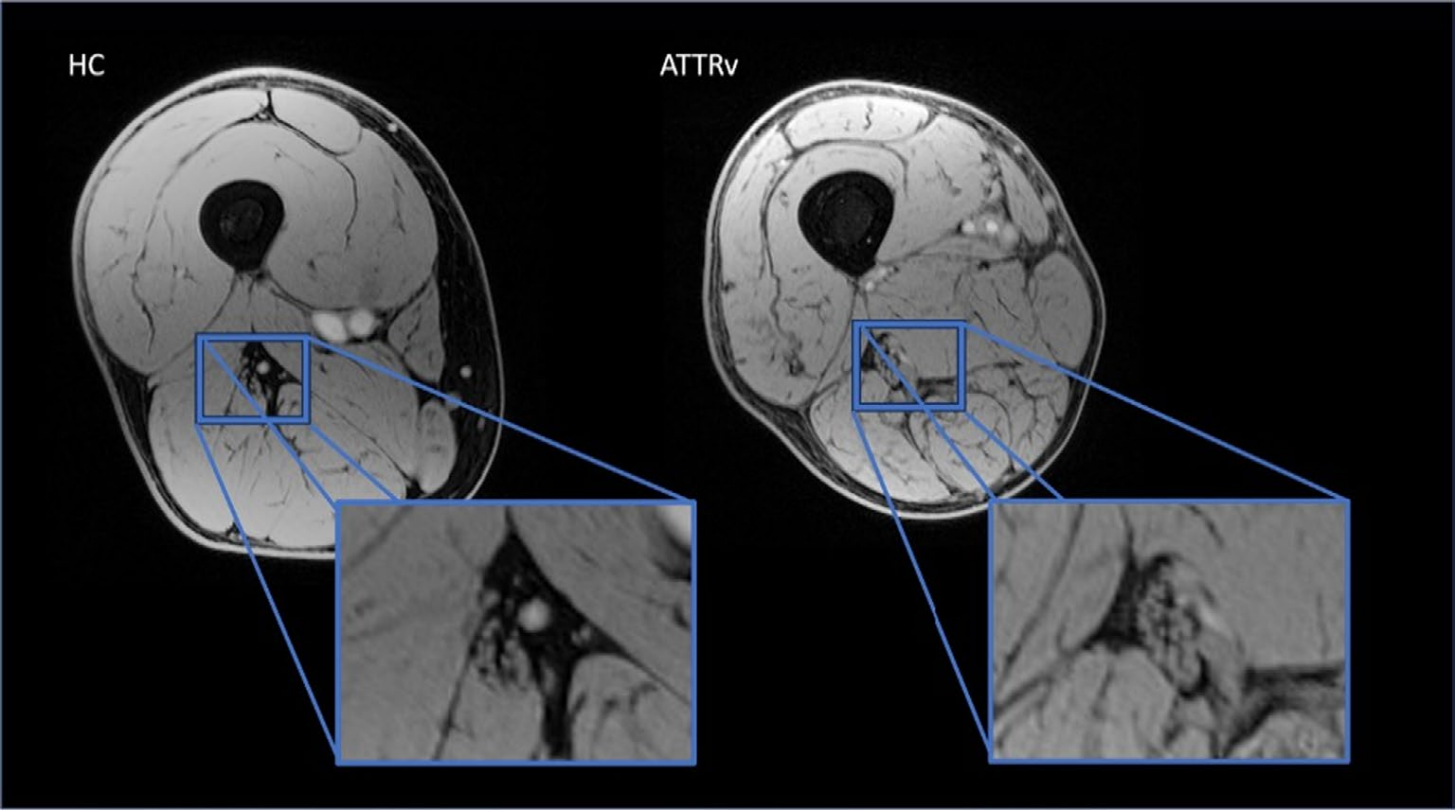
Illustrative image obtained with the vTE sequence on one HC (left) and one ATTRv patient (right), with sciatic nerve magnification allowing the differentiation of the different inner structures of the nerve.

The volume of interest was centered on the right thigh to image the sciatic nerve in the proximal third of the thigh, using a Vitamin E pill placed on the patient's skin as a reference. Figure 1.

For pT2* quantification (ms), the first and last slices of the acquisition were excluded to avoid signal changes due to field inhomogeneities. The central 10 slices were analyzed using ITKSNAP, an open‐source segmentation tool. Two neuroradiologists (C.A. and M.P.), each with 6–8 years of experience, blinded to group and clinical stage, manually drew regions of interest (ROIs) around the whole sciatic nerve on each axial slice. The operators then also segmented the endoneurial fascicles and the inner epineurium within the nerve.

**FIGURE 2:**
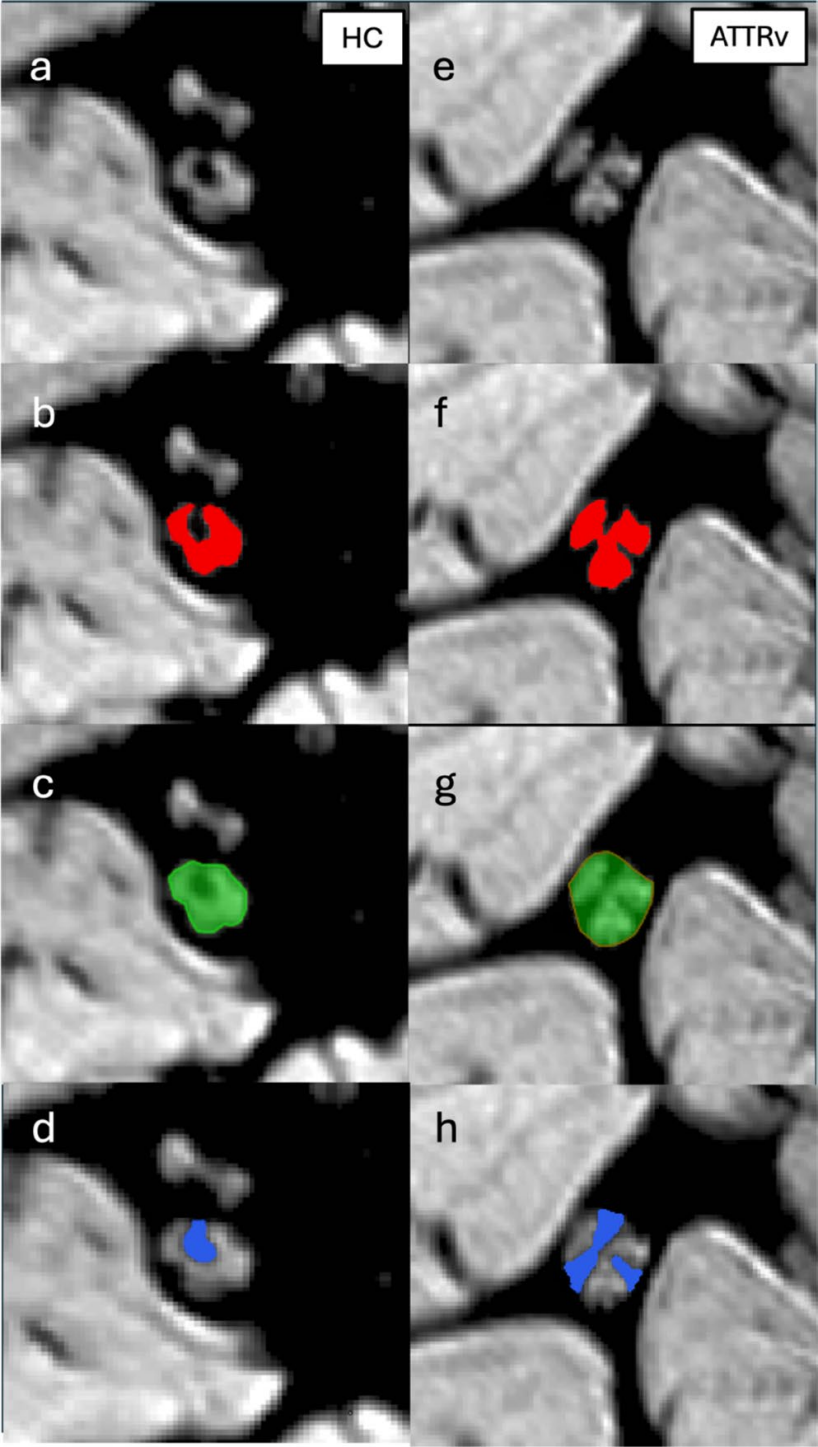
Sciatic nerve images of HC (a) and ATTRv patients (e), with the hyperintense fascicles and the hypointense inner epineurium. Fascicles (b, f), whole nerve (c, g) and epineurium (d, h) segmentation.

Morphometric quantification of sciatic nerve caliber was performed by measuring nerve cross‐sectional area (CSA) on each axial slice, using JIM software (Xinapse, version 8). Additionally, volumetric measures of the fascicular area and inner epineurium area were extrapolated (mm^2^) Figure 2.

The entire segmentation process took approximately 35–40 min per patient. Finally, we calculated the intraclass correlation coefficient (ICC) and the Dice similarity coefficient (DICE) to evaluate inter‐operator reliability [47].

### 2.4 Statistical Analysis

Statistical analyses were performed with SPSS version 22 (IBM, Armonk, NY) with a significance α level of 0.05. For intergroup comparisons, a two‐sample t‐test and Mann–Whitney U test were applied as appropriate. Correlations of MRI parameters with clinical and electrophysiological measures were investigated with Spearman (*ρ*) or Pearson coefficients as appropriate according to data distribution and with general linear mixed‐effects models adjusted for age and sex. A one‐way ANOVA (controls versus mild PNP versus moderate/severe PNP) was performed to analyze the effect of the Group on mean pT2* values. Post hoc pairwise comparisons were performed using the Dunn test to determine specific group differences.

## 3 Results

### 3.1 Description of Patient Sample

We enrolled symptomatic ATTRv patients (*n* = 20) and healthy controls (*n* = 21), matched for age and sex (Table 1). Two out of the 20 patients were excluded from the analyses due to substantial motion artifacts in the MRI images. Eleven/18 (61.1%) patients of the included cohort were males, and the median age at enrollment was 61.6 years (range 43–75), with a median disease duration of 4.5 years (range 2–12).

**TABLE 1:** Demographic and clinical data of ATTRv patients and healthy controls.

Demographics and clinical measures	ATTRv patients (*n* = 18)	Control group (*n* = 22)
Sex, M/F	11/7	14/8
Age, y	61.6 (43–75)	54.6 (42–76)
Median disease duration (range), y	4.5 (2–12)	NA
Treatment, N/tot	15/18	NA
Tafamidis	13/18	
Inotersen	1/18	
Patisiran	1/18	
Mutation		NA
Val30Met	4/18	
Phe64Leu	4/18	
Glu89Gln	2/18	
Tyr78Phe	3/18	
Thr49Ala	1/18	
Ala109Ser	3/18	
Ile68Leu	1/18	
PND‐score		NA
0	NA	
1	10/18	
2	6/18	
3	2/18	
Median NIS (range)	18 (6–170)	
Median NIS‐LL (range)	11 (2–72)	

*TTR* mutations were: Val30Met (*n* = 4, 22.2%), Phe64Leu (*n* = 4, 22.2%), Ala109Ser (*n* = 3, 16.6%), Tyr78Phe (*n* = 3, 16.6%), Glu89Gln (*n* = 2, 11.1%), Thr49Ala (*n* = 1, 5.6%), Ile68Leu (*n* = 1, 5.6%).

The distribution of PND score was the following: PND = 1 (*n* = 10, 55.5%), PND = 2 (*n* = 6, 33.3%), PND = 3 (*n* = 2, 11.1%). The median NIS total and NIS‐LL were 18 (range 6–170) and 11 (range 2–72), respectively. Fifteen/18 (83.3%) patients were on treatment. According to NIS‐LL–based severity classification, 11 patients were diagnosed with mild ATTRv‐PNP (NIS‐LL 1–20), 5 with moderate ATTRv‐PNP (NIS‐LL 21–61), and 1 with severe ATTRv‐PNP (NIS‐LL 62–88) [33]. One/18 patient did not perform clinical assessment at the time of the MRI and was excluded from the statistical analysis. Fourteen/18 (77.7%) patients also agreed to undergo NCS evaluation. Twelve patients showed evidence of purely axonal PNP, whereas 2 patients also showed a reduction in nerve conduction velocity (NCV).

Fourteen/21 (63.6%) HCs were males, and the median age at enrollment was 54.6 years (range 42–76). Demographic, clinical, and genetic data of the participants are summarized in Table 1.

### 3.2 MRI Data

The obtained images had overall sufficient spatial resolution, fat signal suppression, and contrast to correctly visualize the sciatic nerve in all 12 slices of all subjects. The borders and the inner epineurium of the sciatic nerve could be clearly delineated, but the individual fascicles of the nerve could not always be distinguished. Figure 1.

### 3.3 pT2* Signal Quantification

**FIGURE 3:**
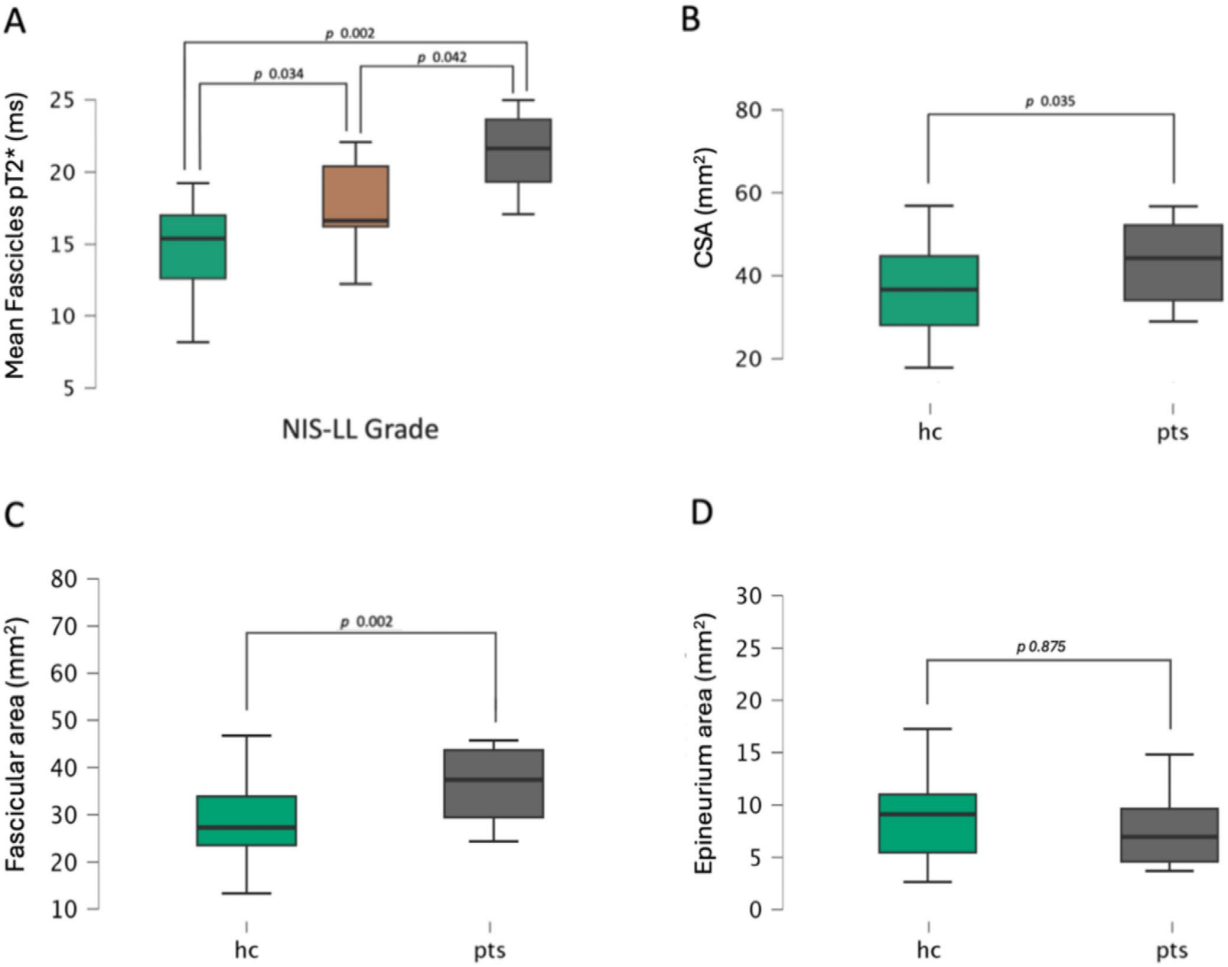
(A) Mean fascicles pT2* values of the right sciatic nerve plotted for HC (green), patients with mild ATTRv‐PNP (brown), and patients with moderate/severe ATTRv‐PNP (gray). Difference in sciatic nerve CSA (B), Fascicular area (C), and Epineurium area (D) between HC and patients.

Sciatic whole nerve and fascicles pT2* values were significantly higher in patients with ATTRv (whole nerve: mean = 15.65 ± 3.04 ms; fascicles: mean = 18.35 ± 3.48 ms) than in controls (whole nerve: mea*n* = 12.64 ± 3.61 ms; fascicles: mean = 14.74 ± 3.21 ms) in a linear regression analysis, controlling for sex and age (whole nerve: *p* = 0.009; *R* = 0.419; fascicles: *p* = < 0.001; *R* = 0.541). No significant differences were found in epineurium pT2* values between patients and HC (*p* = 0.189). Additionally, in the comparison of groups with different degrees of PNP severity (NIS‐LL Grade), fascicles pT2* demonstrated differences among the moderate/severe ATTRv‐PNP (*n* = 6), mild ATTRv‐PN (*n* = 12), and HC groups (*p* = 0.001; *F* value = 8.397). The post hoc comparison revealed a significant increase in fascicles pT2* values within the moderate/severe PNP group (21.33 ± 3.48 ms) when compared to the control group (14.74 ± 3.21 ms; *p* = 0.002). A significant difference was also found between mild ATTRv‐PNP (17.51 ± 3.09 ms) and HC (*p* = 0.034) and between moderate/severe and mild ATTRv‐PNP (*p* = 0.042). Figure 3.

The ICC and DICE values were similar for the whole nerve, fascicles, and epineurium segmentation, indicating excellent reliability (ICC: whole nerve = 0.932, fascicles = 0.912, epineurium = 0.878; DICE: whole nerve = 0.928, fascicles = 0.901, epineurium = 0.889) [47].

### 3.4 Morphovolumetric Assessment

A one‐tail t‐test showed a larger nerve caliber (i.e., CSA) in ATTRv patients than in controls (*p* = 0.017, Patients mean = 49.32 ± 19.63 mm^2^; HC mean = 37.89 ± 12.92 mm^2^). Nerve CSA was significantly larger in mild ATTRv‐PNP groups compared to HC (*p* = 0.017, mild ATTRv‐PNP mean = 53.31 ± 20.37 mm^2^; HC mean = 37.89 ± 12.92 mm^2^).

No significant differences in epineurium area were found between HC (mean = 9.34 ± 5.34 mm^2^) and patients (mean = 7.79 ± 3.61 mm^2^; *F* value = 1.048 *p* = 0.313).

The fascicular area was markedly higher in patients (41.53 ± 16.27 mm^2^) compared to HC (28.91 ± 8.44 mm^2^, *p* = 0.002). We also found a positive correlation between fascicular area and fascicles pT2* (*ρ* = 0.554; *p* < 0.001), with higher pT2* values in nerves with more enlarged fascicles. Figure 3.

### 3.5 Quantitative MRI Parameters Correlate With Clinical Outcomes

We then assessed the role of quantitative vTE sequence as an imaging marker of disease severity by examining its correlation with validated scales of disability (PND‐score) and neurologic impairment (NIS and NIS‐LL).

**FIGURE 4:**
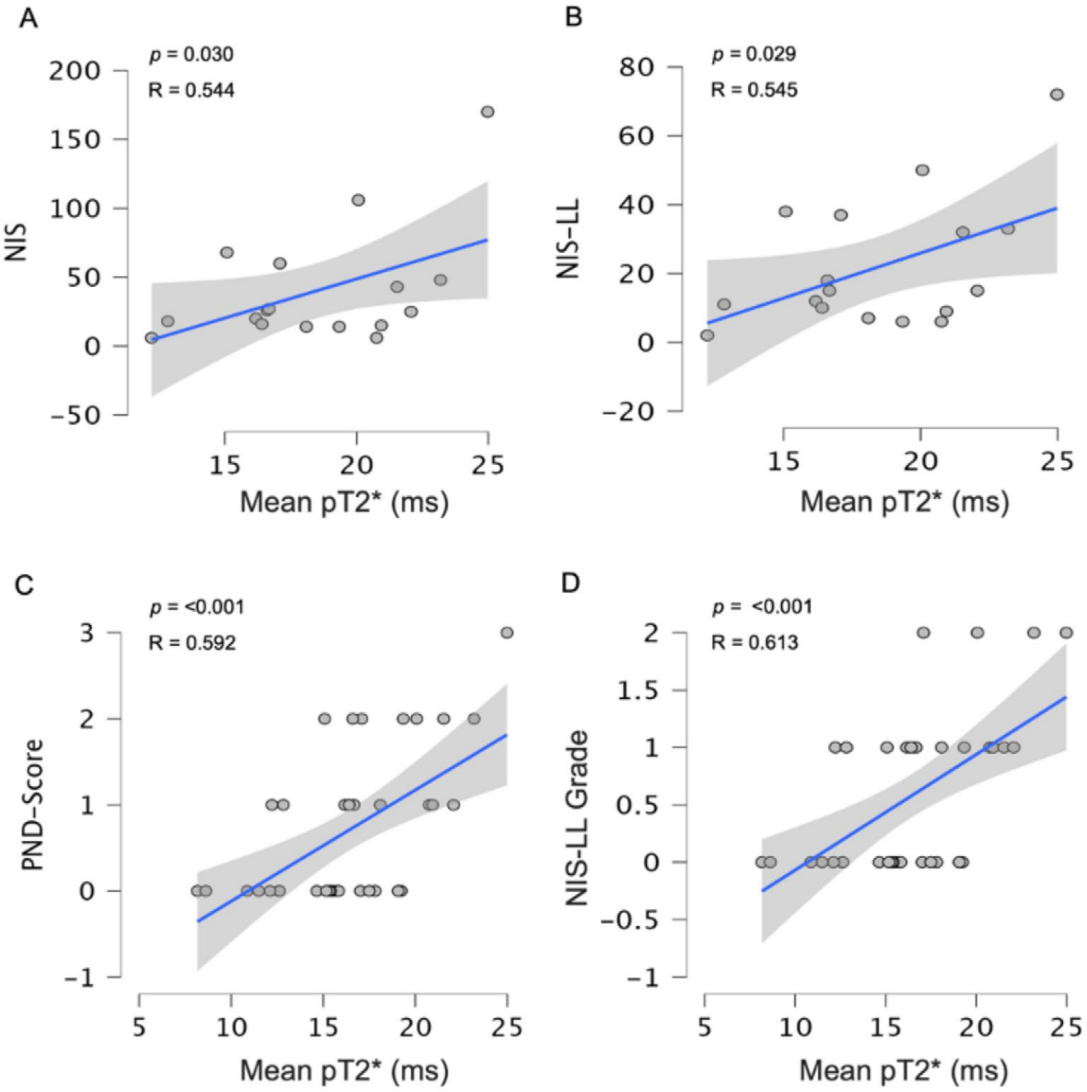
Correlation of pT2* Mean (ms) and NIS (A), NIS‐LL (B), PND‐Score (C) and NIS‐LL Grade (D). pT2* mean values positively correlate with all the validated clinical scales.

A significant association was found between fascicles pT2* values and NIS (*R* = 0.544 *p* = 0.030), NIS‐LL (*R* = 0.545 *p* = 0.032), PND‐score (*R* = 0.592 *p* < 0.001) and NIS‐LL Grade (*R* = 0.613 *p* < 0.001), when controlling for sex and age. Figure 4.

### 3.6 Quantitative MRI Parameters Correlate With NCS Measures

Fascicles pT2* also showed a significant correlation with several electroneurographic parameters. Notably, we observed a negative correlation between fascicles pT2* and CMAP of peroneal (*ρ* = −0.592 *p* = 0.026) and tibial nerves (*ρ* = −0.715 *p* = 0.006). A negative trend between fascicles pT2* and sural SNAP was also found (*ρ* = −0.552 *p* = 0.065).

**FIGURE 5:**
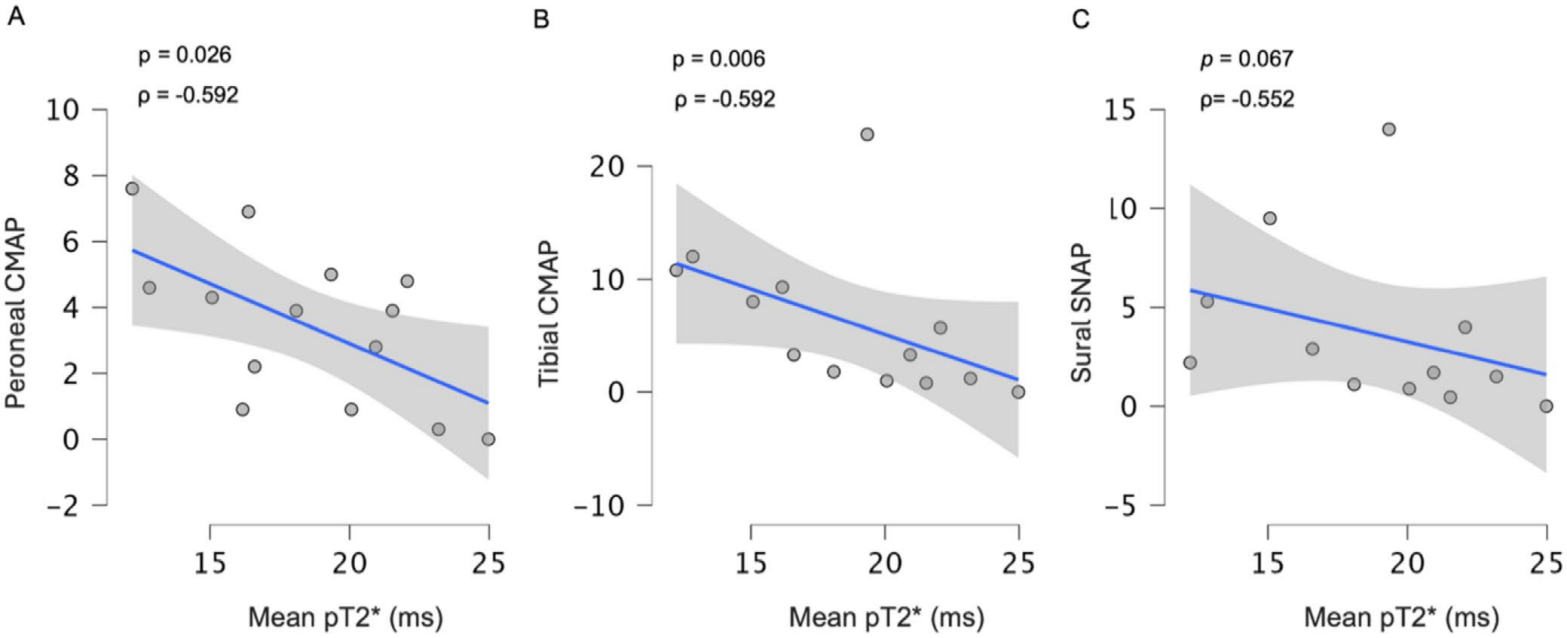
Correlation between Mean pT2* (ms) and nerve conduction parameters of peroneal (A), tibial (B), and sural nerves (C). A significant negative correlation was found with all electrophysiological parameters.

Patients with moderate to severe PNP also showed a reduction (peroneal nerve: *ρ* = −0.519 *p* = 0.042; tibial nerve: *ρ* = −0.531 *p* = 0.038) in nerve conduction velocity (NCV), but with a concomitant reduction in CMAP, suggesting a mixed pattern of axonal and demyelinating damage at a later stage of the disease Figure 5.

## 4 Discussion

In this study, we investigated sciatic nerve involvement in a cohort of ATTRv patients with PNP and showed that the vTE sequence is able to detect pathological changes in the peripheral nerve. In more detail, the main findings of this study are: (i) whole nerve and fascicles pT2* values were increased in ATTRv patients compared to HC, confirming the sensitivity of the vTE sequence to detect pathological microstructural changes within the nerve; (ii) CSA and fascicular area were higher in ATTRv patients compared to HC, whereas the epineurium was only minimally affected; (iii) fascicles pT2* levels reflected disease severity, discriminating mild and moderate/severe PNP from HC; (iv) Lastly, fascicles pT2* correlated with nerve conduction parameters of motor and sensory axonal involvement, appearing as a new promising non‐invasive imaging marker sensitive to peripheral neuropathy.

Peripheral nervous system involvement is a primary manifestation of ATTRv amyloidosis. It is characterized by progressive sensorimotor PNP, which substantially impacts patients' quality of life [1, 2, 3, 4, 5, 6]. Recently, novel RNA‐targeted therapies have demonstrated effectiveness in treating PNP associated with amyloidosis, notably ATTRv amyloidosis, by curtailing abnormal protein formation and stabilizing the normal tetrameric structure of transthyretin [15, 17, 19, 20, 23]. These therapeutic options have remarkably improved the prognosis of ATTRv patients, but timely initiation of treatment remains critical for favorable long‐term outcomes [15, 16, 17, 18, 19, 20, 21, 22, 23]. In this scenario, it is crucial to establish reliable and feasible imaging markers capable of detecting and quantifying the initial peripheral nerve damage associated with amyloid deposition, thereby enabling early‐stage diagnosis and prompt initiation of therapy.

Some previous studies have demonstrated the sensitivity of various quantitative parameters, including NSI, T2app, and proton spin density, in quantifying microstructural changes in the sciatic nerve of ATTRv patients, serving as novel imaging markers for assessing PNS involvement and its progression. Notably, these metrics accurately discriminated patients with ATTRv amyloidosis from HC and had the capability to identify subclinical and early nerve lesions, focusing on the detection of long T2 relaxation time components (unbound water molecules—inflammation) [32, 33, 34]. More recently, both MTR and DTI studies have shown promising and unexpected results. In contrast to the well‐established axonal pattern observed in ATTRv‐PNP, these studies found decreased MTR values and increased RD in DTI analysis within the sciatic nerve of ATTRv patients, both indicating potential myelin involvement [14, 32, 36, 37, 38, 39, 40, 41, 42].

Here we report a study using the vTE sequence to assess sciatic nerve injury in symptomatic ATTRv‐PNP. The vTE sequence enables visualization of the boundaries of the sciatic nerve and differentiation between the fascicles (hyperintense) and epineurium (hypointense). Consistent with previous studies, we observed that CSA was increased in the patient cohort [32, 33, 34]. In this study, we went a step further and extrapolated the fascicular area and the inner epineurium area within the nerve. The increase in CSA and fascicular area observed in patients with overlapping epineurial areas has led us to hypothesize that the larger nerve caliber in the patient group is primarily attributed to an increase in fascicle diameter, while the epineurium appears to remain relatively unaffected [32, 33, 34].

ATTRv patients exhibited a higher sciatic whole nerve and fascicles pT2* values compared to healthy controls, highlighting the sensitivity of the vTE sequence in assessing nerve injury. Due to its very short echo time, this sequence enables the detection and quantification of macromolecular content variations within the nerve, especially within the inner epineurium and fascicles. Prefibrillar material and amyloid fibrils, characterized by a short T2 relaxation time, may also be detected using the vTE sequence [43]. Since the epineurium showed no significant involvement and the fascicular area was larger in ATTRv patients, the observed increase in the sciatic nerve pT2* values may be linked to microstructural alterations primarily occurring within the fascicles. These findings align with autopsy and histopathological studies showing that the endoneurium is the most affected inner structure of the nerve in the pathological process of ATTRv‐PNP [13]. Higher pT2* values in fascicles of ATTRv patients could be attributed to variations in myelin content or to the accumulation of substances with short‐ultrashort T2 times, such as prefibrillar material and amyloid fibrils. However, given the inverse relationship between the content of short T2 time components and pT2* values, amyloid deposition would likely lead to a decrease in pT2*. Therefore, a possible reduction in myelin content in the fascicles as the primary factor driving the observed increase in pT2* cannot be excluded.

These findings are supported by postmortem and biopsy studies wherein areas of myelin loss have been consistently observed in large myelinated fibers, particularly at sites of amyloid deposition. This leads to cytoplasmic degenerative changes within Schwann cells, resulting in a reduction of myelin content in myelinated fibers and of small unmyelinated fibers primarily due to the decreased trophic support [10, 47].

Similar to the other quantitative measures mentioned above, pT2* reflects clinical severity and correlates with all validated clinical outcome measures of ATTRv‐PNP (PND‐score, NIS, NIS‐LL). Interestingly, the vTE sequence can differentiate patients with mild ATTRv‐PNP from HC by detecting microstructural changes in the early stages of nerve damage, thus potentially helping the clinical decision in the initiation of therapy.

Being negatively correlated with CMAP values of peroneal and tibial nerves in ATTRv‐PNP patients, pT2* appears to reflect the severity and extent of motor axonal loss and/or degeneration. This is clinically relevant because pT2* is measurable and, therefore, applicable for disease monitoring in even more advanced stages of PNP when CMAPs are no longer elicitable [39]. According to previous studies, we also found a reduction in conduction velocity in the later stages of the disease (moderate to severe), confirming the presence of a microstructural pattern distinct from other purely axonal polyneuropathies [32, 39]. If myelin involvement is present, especially in large deep nerves, further investigation using alternative approaches is warranted. It is important to note that the most common misdiagnosis of ATTRv‐PNP is chronic inflammatory demyelinating polyradiculoneuropathy (CIDP) [32, 39].

Our study has some limitations. First, the sample size was limited, and we recruited mostly ATTRv patients with mild to moderate neuropathy, while more advanced stages of the disease were underrepresented. Nerve biopsy was not performed in any of the cases, so a radiological‐histological correlation was not possible. In addition, the anatomical coverage of the vTE acquisition is relatively small compared to the length of the peripheral nerve and does not allow assessment of differences along the nerve, considering that most peripheral neurological diseases have different patterns of nerve involvement. The sciatic nerve was analyzed in the proximal thigh without further evaluation of tibial and peroneal divisions. Only the right thigh was examined, in line with several previous studies that already focused on one leg, as no significant differences were found between the two legs in ATTRv patients [32, 33, 39]. Additionally, the segmentation of the endoneurial fascicles also included a portion of the inner epineurium, which may have influenced the effective pT2* values extrapolated from the fascicles. Finally, a multi‐echo approach, with short‐ultrashort TE, may have been more suitable for detecting the microstructural changes that occur within the nerve in relation to amyloid deposition, providing a more precise and sensitive measure of nerve damage.

In conclusion, the vTE MRI sequence proved to be suitable for the detection and quantification of short T2* nerve components in our ATTRv patients, including myelin components, providing a potential imaging marker of nerve damage related to the deposition of aberrant proteins. The derived measure (i.e., pT2*) also reflected disease severity and eventually succeeded in detecting nerve injury during the early disease stages in patients with mild PNP. Longitudinal studies are warranted to assess the role of vTE as a noninvasive, objective, and sensitive imaging marker for the diagnosis and monitoring over time of ATTRv‐PNP patients, especially in pre‐symptomatic and early symptomatic stages of the disease.

## Author Contributions

Carlo Asteggiano: study concept and design, acquisition of data, analysis and interpretation of data, statistical analysis, study supervision and coordination, writing the manuscript. Matteo Paoletti: acquisition of data, analysis and interpretation of data, critical revision of manuscript for intellectual content. Elisa Vegezzi: acquisition of data, analysis and interpretation of data, critical revision of manuscript for intellectual content. Xeni Deligianni: analysis and interpretation of data, critical revision of manuscript for intellectual content. Francesco Santini: analysis and interpretation of data, critical revision of manuscript for intellectual content. Niels Bergsland: analysis and interpretation of data, critical revision of manuscript for intellectual content. Nico Papinutto: analysis and interpretation of data, statistical analysis, critical revision of manuscript for intellectual content. Massimiliano Todisco: acquisition of data, analysis and interpretation of data, critical revision of manuscript for intellectual content. Giuseppe Cosentino: contribution of patients, critical revision of manuscript for intellectual content. Andrea Cortese: study concept and design, acquisition of data, analysis and interpretation of data, critical revision of manuscript for intellectual content. Laura Obici: acquisition of data, critical revision of manuscript for intellectual content. Giovanni Palladini: acquisition of data, critical revision of manuscript for intellectual content. Anna Pichiecchio: study concept and design, acquisition of data, study supervision and coordination, critical revision of manuscript for intellectual content.

## Conflicts of Interest

The authors declare no conflicts of interest.

## Data Availability

Anonymized data not published within this article are available in the Zenodo repository at https://doi.org/10.5281/zenodo.14198626.
